# Deep learning architectures for prediction of nucleosome positioning from sequences data

**DOI:** 10.1186/s12859-018-2386-9

**Published:** 2018-11-20

**Authors:** Mattia Di Gangi, Giosuè Lo Bosco, Riccardo Rizzo

**Affiliations:** 1 Fondazione Bruno Kessler, Via Sommarive, 18, Trento, 38123 Italy; 2 ICT International Doctoral School, Via Sommarive, 9, Trento, 38123 Italy; 3 Dipartimento di Matematica e Informatica, Università degli studi di Palermo, Via Archirafi, 34, Palermo, 90123 Italy; 4 Dipartimento di Scienze per l’Innovazione tecnologica, Istituto Euro-Mediterraneo di Scienza e Tecnologia, Via Michele Miraglia, 20, Palermo, 90139 Italy; 5 CNR-ICAR, National Research Council of Italy, Via Ugo La Malfa, 153, Palermo, 90146 Italy

**Keywords:** Nucleosome classification, Epigenetic, Deep learning networks, Recurrent neural networks

## Abstract

**Background:**

Nucleosomes are DNA-histone complex, each wrapping about 150 pairs of double-stranded DNA. Their function is fundamental for one of the primary functions of Chromatin i.e. packing the DNA into the nucleus of the Eukaryote cells. Several biological studies have shown that the nucleosome positioning influences the regulation of cell type-specific gene activities. Moreover, computational studies have shown evidence of sequence specificity concerning the DNA fragment wrapped into nucleosomes, clearly underlined by the organization of particular DNA substrings. As the main consequence, the identification of nucleosomes on a genomic scale has been successfully performed by computational methods using a sequence features representation.

**Results:**

In this work, we propose a deep learning model for nucleosome identification. Our model stacks convolutional layers and Long Short-term Memories to automatically extract features from short- and long-range dependencies in a sequence. Using this model we are able to avoid the feature extraction and selection steps while improving the classification performances.

**Conclusions:**

Results computed on eleven data sets of five different organisms, from Yeast to Human, show the superiority of the proposed method with respect to the state of the art recently presented in the literature.

## Background

The genome of eukaryote species is embedded into the nuclei of their cells. This is due to the presence of *nucleosomes*, which are histone octamers where 150 bp of DNA are wrapped in about 1.7 turns, separated by stretches of DNA referred as *linker* sequences. Starting from this low-level organization, the nucleosomes arrange themselves through successively higher-order structures to finally form the chromosomes. Apart from this packing property, the genome-wide location of the nucleosomes is fundamental for many biological processes. Gene regulation is maybe the most relevant of these processes, established by the nucleosome property of influencing DNA protein binding. For example, several studies have shown evidence that the transcription activity in promoter regions is inversely proportional to the nucleosome lacking [1]. Other important biological problems directly related to nucleosome positioning are co-transcriptional splicing, DNA replication and DNA repair [2–5].

A more detailed characterization of the eukaryotic DNA machinery is that nucleosome positioning and high-order chromatin structures together constitute a sort of control logic of this machinery. Moreover there is special evidence that distinct DNA sequence features are associated to nucleosome presence [6, 7]. Unfortunately, this is not the unique cause that determines the phenomenon, as demonstrated by the influence that special proteins can operate to nucleosomes [8]. Nonetheless, there is a special interest in understanding to which extent the DNA sequence specificity is responsible for nucleosome positioning. This is demonstrated by the number of computational models that have been proposed so far [9]. Some of them are based on statistical models that use as a priori information the estimated distributions of dinucleotides from in vitro and in vivo sequences [10–13]. Also biophysical inspired models have been proposed [14, 15]. Others use machine learning methodologies such as logistic regression [16], Hidden Markov Models [17] and support vector machines [18]. The main characteristic shared by all the above-mentioned methodologies is the use of manually-extracted features to represent the input sequences. Establishing such details is crucial for the effectiveness of the computational solution, and this task is usually performed by supervision of the experts and a preliminary step of feature representation.

For example, in the case of the machine learning methodology for nucleosome identification, an effective way of representing the sequences is by the so called *k-mers* representation as demonstrated by several methodologies [19–25]. This representation maps a DNA sequence into a numerical space by means of a fixed-length vectors whose components are the count of each of the substrings belonging to a finite set of words.

Among the possible machine learning methods, the recent adoption of the so called *deep learning neural networks* (DLNN) [26] has produced as result very important advancements in several arduous artificial intelligence tasks [27]. We can affirm that nowadays, DLNN represents the state of the art for most of the machine learning problems. Moreover, another special property of the DLNN models that contributes to further improve their potentiality is the so called *representation learning property* i.e. the ability of such methods to automatically extract and consider useful features from the input patterns without using any a priori knowledge about the problem domain.

The two most effective kinds of DLNN successfully adopted so far for sequential input data belong to the classes of *Convolutional Neural Networks* (CNNs) and *Recurrent Neural Networks* (RNNs). CNNs have a feedforward scheme, and are mainly composed of an initial layer of convolutional filters whose output is processed by nonlinear activation functions followed by a sub-sampling layer. The final classification is decided by a fully connected layer [28]. Collobert et al. [29] firstly shown that CNNs can be used effectively also for sequence analysis, in the general case of text classification.

RNNs have been proposed for processing sequential data. They are characterized by an hidden state that acts like an internal memory. The memory about the past is implemented by feedback connections directed through the hidden state so that it can be updated taking into consideration what happened in the previous time steps. Due to this memory property, RNNs are able to handle sequence information over time, and this could represent a prominent property for sequence classification.

Some DLNN for sequence classification have been proposed so far, both for generic sequences [30–32] and for the case of nucleosome related ones [33, 34]. In particular, a CNN architecture that adopts the k-mers as feature representation and extraction step has been proposed for nucleosome classification[33]. Moreover, a more effective version of a DLNN which uses a convolutional layer for automatically extract the sequence features, and a recurrent layer to deal with the longer-range positional dependency between part of the sequence has been recently proposed [34].

In this work, we extend this latter contribution by slightly modifying the network architecture and enlarging the experimental part with new datasets and more evaluation setups. The extended evaluation allows a comparison of the proposed methodology for nucleosome identification against many previously proposed methodologies. The main advantage of the proposed method is that it does not need any feature extraction or selection process, a step that seems to be fundamental for all the most successful computational methods for nucleosome identification.

## Methods

DLNNs represent now the state of the art approach for many machine learning applications: they are characterized by the presence of several stacked layers, each producing a higher-level representation of the data coming from the previous layer. For a classification task, the last layer is able to predict the probabilities for each class relying on a high-level representation of the data that has been learnt automatically by the network. One interesting aspect of deep learning is its capability to learn to extract features starting from input in a raw form, i.e. one-hot encoding for genome sequences, even though it’s possible to add external knowledge from which the network can benefit. In this work, we do not take in consideration the latter option. In the deep learning literature, we can find various kinds of layers that can be applicable to different types of data and can be chosen according to the type of features we expect can be useful for the task. For instance, convolutional layers can learn local relations in a sequence or an image, recurrent layers can learn features useful for sequences of data and the more classical feed-forward layers can learn global features from the samples.

A network architecture is given by the number, the kind and the sequence of the layers, together with the parameter values.

In the following subsections we will introduce the basic layers used to build our neural model and will discuss the architecture used for the network, finally, we will describe the datasets used for the experiments.

### Convolutional layer

In general, a 1-D convolutional layer processes an input *x* in the form of numerical matrix of size *m*×*R*, giving as output a matrix *h* of size *L*×*R*, where *L* is the number of output feature maps. A feature map *h* is the result of the convolution of the data and a kernel *w*^*l*^ of length *k*, to which a nonlinear function *g* is applied:

1 $$ q^{l}_{i}=\sum\limits_{u=0}^{k} \mathbf{w}^{l}(u)\mathbf{x}(i-u)\\   $$

2 $$ h^{l}_{i}=g\left(q^{l}_{i}+b^{l}\right)   $$

In the formulas above, *k* is a parameter of the convolution and represents the number of adjacent string positions taken into account during the convolution. The choice of *k* determines the kind of features that can be learned by the network, as well as the computational speed. A higher value of *k* means that a larger area is covered each time, but requires more computations to be performed. Common values of *k* range from 1 to 7. We choose to use filters of size not greater than 5 because the output of the convolutional layer can be seen as continuous vectors for *k*-mers, and from the previous study seems that *k*-mers longer than 5 do not help in this classification task.

In many cases, a convolutional layer is followed by a pooling layer that is used to subsample and regularize the input, in order to speed up the computation process and to prevent overfitting. The *max-pooling* acts as a nonlinear down-sampling by partitioning the input vector into a set of non-overlapping regions and, for each sub-region, their maximum value is chosen as output. This processing layer reduces the complexity of the following layers and operates a sort of translational invariance. This characteristic can be useful in the case of DNA sequences, in particular when the presence of certain DNA substring is more relevant than their positioning.

### Long short-term memory layer

A recurrent neural network (RNN) is a processing unit capable to process sequences of data. It stores a so-called *hidden state* that is updated after the computation of every time step. The new hidden state is a function of the old hidden state and the input at the current time step:

$$\mathbf{h_{t}} = g\left(\mathbf{W_{h}} \mathbf{x_{t}} + \mathbf{U_{h}} \mathbf{h_{t-1}} + \mathbf{b_{h}}\right)$$

where *W*_*h*_ and *U*_*h*_ are respectively the weight matrices for the input and the hidden state, *b*_*h*_ is a bias vector, and *g* is a nonlinear activation.

For each time step *t*, the hidden state *h*_*t*_ contains a summary of the sequence seen until time step *t*, thus the last hidden state *h*_*T*_ should contain a summary of the sentence. Unfortunately, as *W*_*h*_ and *U*_*h*_ are fixed during the processing of a sequence, the importance of each input decays exponentially with the distance from the end. Another problem that limits the learning capability of this kind of layer is the *vanishing gradient problem* [35], which greatly reduces the number of time steps that are actually involved in the learning. A *Long Short-Term Memory* (LSTM) layer [36] is a variant of a recurrent layer explicitly designed to alleviate the vanishing gradient problem, and selecting the inputs that are relevant for updating the hidden state. The LSTM can be described by the following equations

$$\mathbf{f_{t}} = \sigma\left(\mathbf{W}_{\mathbf{f}}\mathbf{x}_{\mathbf{t}} + \mathbf{U}_{\mathbf{f}}\mathbf{h}_{\mathbf{t-1}} + \mathbf{b}_{\mathbf{f}}\right) $$

$$\mathbf{i}_{\mathbf{t}} = \sigma\left(\mathbf{W}_{\mathbf{i}}\mathbf{x}_{\mathbf{t}} + \mathbf{U}_{\mathbf{i}}\mathbf{h}_{\mathbf{t-1}} + \mathbf{b}_{\mathbf{i}}\right) $$

$$\mathbf{o}_{\mathbf{t}} = \sigma\left(\mathbf{W}_{\mathbf{o}}\mathbf{x}_{\mathbf{t}} + \mathbf{U}_{\mathbf{o}}\mathbf{h}_{\mathbf{t-1}} + \mathbf{b}_{\mathbf{o}}\right) $$

$$\mathbf{c}_{\mathbf{t}} = \tanh\left(\mathbf{W}_{\mathbf{c}}\mathbf{x}_{\mathbf{t}} + \mathbf{U}_{\mathbf{c}}\mathbf{h}_{\mathbf{t-1}} + \mathbf{b}_{\mathbf{c}}\right) $$

$$\mathbf{s_{t}} = \mathbf{f_{t}} \odot \mathbf{s_{t-1}} + \mathbf{i_{t}} \odot \mathbf{c_{t}} $$

$$\mathbf{h_{t}} = \tanh\left(\mathbf{s_{t}}\right) \odot \mathbf{o_{t}}\\ $$

where *i*_*t*_,*f*_*t*_,*o*_*t*_ are respectively, the input, forget and output gates, and ⊙ represents the element-wise multiplication. The function sigma and tanh indicate sigmoid and hyperbolic tangent functions respectively. The gates are vectors that assume values in the range [0,1] and decide, component by component, which part of the input, of the previous hidden state and of the candidate output should flow through the network.

The LSTMs are used nowadays for several applications ranging from automatic speech recognition [37], machine translation [38], image captioning [39], and all the tasks requiring sequence processing or generation.

### Fully connected layer

The fully connected layer is constituted by arrays of nonlinear units where each unit calculates a weighted sum of all the outputs from the preceding layer and generates an output signal by using a nonlinear function. The nonlinearity can be a smooth curve as the sigmoid or a piecewise linear function as the so-called *rectified linear units* (ReLU).

### Dropout

The dropout [40, 41] is a layer that acts as a regularization to prevent the overfitting. It can be added after any layer of the network and it randomly sets to zero units from the preceding layer with a fixed probability *p* during the training. Without the dropout, each computing unit is pushed to learn to detect one single feature from the preceding layer, which is a clear sign of overfitting. By shutting down the units randomly, the network cannot rely on the outcome of specific units and the learning is spread among the features. The regularization obtained with this method can be considered as an efficient model averaging. In fact, as during each round of training the network architecture is different, it is equivalent to train a high number of networks with shared parameters. When *p*=0.5, it is equivalent to training 2^*W*^ networks with shared parameters, where *W* is the number of neurons subject to dropout.

### The proposed deep architecture

Finding the most suitable architecture for a DLNN is an open problem. Zeng et al. [42] analyze the design of the convolutional neural networks for sequence classification. The authors studied the structure of the convolutional neural network by varying the number of kernels and the number of layers. Among the conclusions of the work, the authors find that increasing the number of convolutional layers gives only a small improvement in the network performances, and a more complex architecture needs more training time and more training data to obtain a little gain in the classification results. Starting from these observations, the authors conclude that CNN performances do not scale easily with the complexity of the network. Following these results, in this work we experiment a different approach by adding an LSTM layer after the convolution and the sub-sampling. The idea is to let the convolutional layer to extract the most relevant local features, and in the following step use the LSTM to find the relations of these features along the sequence.

**Fig. 1:**
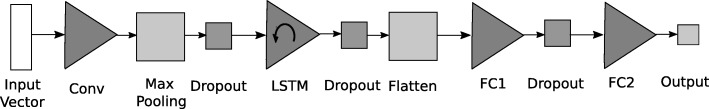
The deep neural network architecture of the classifier

The proposed architecture is composed of different layers, as it is shown in Fig. 1: a convolutional layer, a max pooling layer, a dropout layer, a LSTM layer, and two fully connected layers. The first layer from left to right is a convolutional layer whose main role is the feature extraction from the input data *x* of 4×*R* binary values, where *R* is the length of the sequence. This layer is composed by a bank of *n*=50 1*D* convolutions [28] between the kernel vectors *w*^*l*^ *l*=1,2,…*n* and the input sequence *x*. The subsequent Max Pooling layer with width and stride 2 helps to capture the most salient features extracted by the convolution and reduces the output size of the input vectors. Then, the Dropout operation with probability *p*=0.5 is used to prevent overfitting during the training phase. The LSTM layer scans the sequential features output of the previous layer and outputs its hidden state at each time step, it has 50 hidden memory units. The purpose is to find long-range relations between the time steps along all the sequence. The convolutional and LSTM layers have a regularizer *L*_2_ with *λ*=0.001. The outputs from all the LSTM time steps are then concatenated in a single vector. This vector is then fed to 2 subsequent fully-connected layers that reduce its length to 150 and then to 1. The first fully connected layer has a *ReLU* nonlinearity, and the output of the network is a real value in the interval [0,1] calculated by using a sigmoid function. We recall that the model we propose does not need any prior information about feature representation by means of any feature engineering process. For this reason, the input of our model is in the form *character-level one-hot encoding*. This representation takes into account each character *i* of the alphabet {*A*,*C*,*G*,*T*} by a vector of length equal to the alphabet size, having all zero entries except for a single one in position *i*. This method leads to a sparse representation of the input.

The network has been implemented using the Keras [43] environment on different hardwares running both Tensorflow [44] and Theano [45] libraries deep learning framework, and it has been trained by stochastic gradient descent using the *Adam* optimizer [46]. The loss function is the binary cross entropy, which makes the training of the last layer equivalent to a logistic regression, but with the back-propagating gradient that affects also the feature learning.

### Dataset descriptions

In this work we have used two groups of datasets, which are related to two recent papers about nucleosome positioning identification methods [18, 47]. We have chosen these datasets because they have been used to evaluate at least one state-of-the-art methodology for nucleosome positioning identification. As a consequence, we can easily put our results in comparison with several recently proposed methods.

The first group of datasets is composed of three sets of DNA sequences underlying nucleosomes from *Homo sapiens*(HM), *Caenorhabditis elegans* (CE) and *Drosophila melanogaster*(DM). The details about all the steps that have been adopted in order to extract and filter such data can be found in the paper by Guo et al. [18] and in the references therein. Each dataset is composed of data sequences of length 147 base pairs (bp) grouped in two classes of samples: the nucleosome-forming sequence samples (positive data) and the linkers, or nucleosome-inhibiting sequence samples (negative data). The distribution of the classes for this group of datasets is shown in Table 1. The second group of datasets is related to the paper by Liu et al. [47] and is composed of eight sets of DNA sequences related to three species: *Homo sapiens* (HM), *Drosophila melanogaster* (DM) and *Yeast* (YS). Unlike the first group of datasets, in this second case, different categories of sequences are present from each of the species. In particular, we find whole genome (WG) and promoter (PM) sequences of YS, and the largest chromosome (LC), promoter (PM) and 5’UTR exon region (5U) sequences from DM and HM. The authors of the related paper have provided only the bed files of the datasets, thus we had to retrieve the sequences of each set with the following procedure. We have first extracted the coordinates of the midpoints of each nucleosomal and linker sequence from the bed files and got the sequence range coordinates by extending 75 bp left and 75 right of the midpoint. We have then fetched the 151 bp sized nucleosomal and linker sequences using the genome files downloaded from UCSC Table Browser. The distribution of the classes for this group of datasets is shown in Table 2.

**Table 1:** The distribution of samples in the first group of dataset

	First group		
	HM	DM	CE
N	2273	2900	2567
L	2300	2850	2608
T	4573	5750	5175

HM indicates the group of Human sequences; DM indicates the group of Drosophyla sequences; CE indicates the group of C. Elegans sequences. The row label N indicates the nucleosome sequences; the row label L indicates the linker sequences and T inidicates the total number of sequences

**Table 2:** The distribution of samples in the second group of dataset

	Second group							
	HM			DM			YS	
	LC	PM	5U	LC	PM	5U	WG	PM
N	97209	56404	11769	46054	48251	4669	39661	27373
L	65563	44639	4880	30458	28763	2704	4824	4463
T	162772	101043	16649	76512	77014	7373	44485	31836

The meaning of the row labels is the same of the Table 1; The label YS indicates the Yeast group of sequences; WC indicates the whole genome, LC indicates the largest chromosome, PM indicates the Propoter sequences; 5U indicates the sequences from the 5UTR exon region

## Results an discussion

We have performed experiments in two different settings, one for each group of datasets, in order to be comparable with the state of the art.

In the first setting, a 10-fold schema is used to evaluate the first dataset group. For each iteration, 1 fold is used for testing and the other 9 for training. A 10% of the dataset is selected among the training set as a validation step for early stopping. The predicted labels are obtained by thresholding the output value of the DLNN, that ranges in the interval [0,1], with the value 0.5. Output values below 0.5 are classified as linkers otherwise as nucleosomes. In the second experimental setting, we have computed the receiver operating characteristic (ROC) curves and the Area under the ROC curves (AUC) for all the datasets of the second group. Liu et al. [47] have proposed an evaluation protocol for these datasets that consists in sampling 100 test samples (with replacement) of 100 sequences each from the whole dataset, computing the ROC curve for every sample and finally the average of the sensitivity and specificity over the 100 samples. Unlike the methods used in that work, our method requires a training set; hence we have split each dataset a priori in a training, validation and test set. The split between training and test set is made in a way that there are enough data for training a strong model while having a large pool from which to select test data, and not to bias the comparison. After the training/test split, we select a number of samples equivalent to the 10% of the dataset size from the training set as a validation set used for early stopping. If the validation size would exceed 1000, we clip it to this number. The numbers used for the splits are listed in Table 3. The training phase is constituted by 100 epochs, with a learning rate of 3∗10^−4^. The inputs are divided in min batches with a batch size of 64. For each dataset we train one only model using the selected training set and then compute the ROC curve and the AUC, as well as the specificity and sensitivity over the 100 tests. Specificity and sensitivity are computed by setting a score threshold, i.e. a sequence is classified as a nucleosomal sequence if the corresponding output of the network is greater than the predefined threshold or otherwise it is classified as a linker sequence. For all the experiments we have used two different networks, named *DLNN-3* and *DLNN-5*. The two networks differ in the size of the convolution kernels that compose the first layer of the network, which is 3 for the first network and 5 for the second.

**Table 3:** Training/Validation/Test split for the second group of datasets

Dataset	Total samples	#Training	#Validation	#Test
HM-LC	162772	81298	1000	80474
HM-PM	101043	51321	1000	48722
HM-5U	16649	10654	1000	4995
DM-LC	76512	55512	1000	20000
DM-PM	77014	68312	1000	7702
DM-5U	7373	3687	737	2949
YS-WG	44485	39485	1000	4000
YS-PM	31836	27836	1000	3000

### Results on first group of dataset

The results obtained by the DLNN we propose are shown in Table 4. We have reported mean values of accuracy, sensitivity and specificity of the three datasets computed by the two versions of the DLNN. We also report the results of the *iNuc-PseKNC* method computed on the same dataset as shown in the results of the related paper [18]. In the table, the best values are shown in bold. It is clearly observable that the models we are proposing outperform the iNuc-PseKNC on each dataset in terms of Sensitivity. This means that our models are more able to predict nucleosome class than iNuc-PseKNC. The improvements in the sensitivity of our methods are always considerable and can be up to +9*%* for the case of DM. The method iNuc-PseKNC continues to be better than our model in accuracy and specificity only on the HM dataset.

**Table 4:** 10-fold cross validation performances on the first group of dataset

Method(Species)	Accuracy		Sensitivity		Specificity	
	*μ*	*σ*	*μ*	*σ*	*μ*	*σ*
iNuc-PseKNC(CE)	86.90	x	90.30	x	83.55	x
iNuc-PseKNC(DM)	79.97	x	78,31	x	81.65	x
iNuc-PseKNC(HM)	**86,27**	x	87,86	x	**84,70**	x
DLNN-3(CE)	**89.60**	0.8	**93.36**	1.27	**85.93**	2,13
DLNN-3(DM)	**85.54**	1.13	**87.60**	2.55	**83.42**	2.65
DLNN-3(HM)	84.65	2.16	**89.67**	2.83	79.64	4.29
DLNN-5(CE)	**89.62**	2.45	**93.04**	3.68	**86.34**	5.54
DLNN-5(DM)	**85.60**	0.75	**87.81**	2.79	**83.33**	2.74
LNN-5(HM)	85.37	1.91	**88.34**	1,82	82.29	4.86

iNuc-PseKNC refers to the method introduced in [18]; CE, DM, HM refers to the datasets descried in Table 1; DLNN refers to the DLNN proposed in this paper and -3 or -5 refers to the kernel dimension in the first convolutional layer of the net. Best values are in bold

### Results on second group of dataset

On these group of datasets, we know the results of eight state of the art methodologies for nucleosome positioning identification [10–17] as reported in the reference paper [47]. Table 5 summarize the AUCs computed by other methods in the first eight column of the table. Note that in most of the cases the paper by Liu [47] does not report precise values of the AUC, thus we have reported approximate values in terms of open interval (minimum and maximum values of the interval in brackets) or very close to (symbol ’ ∼’). Such approximate values have been extracted looking at the plot bars shown in the paper. The last two column summarizes the experiments of the two version of the networks (DLNN-3 and DLNN-5). We point out that the networks share the same architecture, but use different training set opportunely extracted from the species, and complementary to the test set used for computing the ROC. Also in the case of this kind of experiments, the bold values indicate the best AUC performances. In the case of DLNN-3, its AUC values are better than any other method on HM-LC, YS-WG, HM-PM datasets, and equal to the best on DM-LC, DM-PM, DM-5U. Only in the case of YS-PM and HM-5U the AUC values are worst but very close to the best ones. The AUC values are further improved by the DLNN-5, showing the best values on HM-LC, YS-WG, HM-PM, YS-PM, and performing equally to the best in DM-PM, DM-LC, letting HM-5U the only dataset where it performs worst.

**Table 5:** Area under the ROC curve performances on the first group of dataset

	N_score [16]	NuPop [17]	NucEnergEN [14]	Segal [10]	Field [11]	Kaplan [12]	Heijden [13]	Finestr [15]	DLNN-3	DLNN-5
HM-LC	∼0.65	(0.6,0.65)	(0.6,0.65)	(0.35,0.4)	∼0.65	∼0.65	∼0.6	(0.6, 0.65)	**0.79**	**0.81**
DM-LC	0.59	(0.65,0.70)	∼0.7	0.33	(0.70,0.75)	**(0.70,0.75)**	(0.65,0.70)	0.57	**0.71**	**0.71**
YS-WG	0.77	0.74	(0.65,0.70)	0.49	0.77	∼0.7	∼0.65	∼0.7	**0.79**	**0.83**
HM-PM	(0.6,0.65)	0.67	(0.6,0.65)	(0.4,0.45)	(0.6,0.65)	∼0.6	∼0.55	∼0.55	**0.77**	**0.77**
DM-PM	0.62	∼0.7	(0.70,0.75)	0.32	**(0.70,0.75)**	**(0.70,0.75)**	(0.55,0.6)	(0.5,0.55)	**0.72**	**0.73**
YS-PM	0.70	0.74	(0.7,0.75)	0.52	0.79	(0.7,0.75)	∼0.65	∼0.7	0.73	**0.83**
HM-5U	(0.55,0.6)	∼0.65	**∼0.7**	0.37	∼0.65	∼0.65	∼0.6	(0.55,0.6)	0.67	0.68
DM-5U	0.54	(0.6,0.65)	(0.65,0.70)	0.38	**(0.65,0.70)**	**(0.65,0.70)**	(0.55,0.6)	∼0.5	**0.66**	**0.67**

Each column refers to a computational method for nucleosome positioning. The rst eight column show the values reported in the paper by Liu et al. [47], sometimes with approximate values (interval range or close to symbol”). Last two columns regard our proposed method. Best values are in bold

## Conclusions

In this study, a deep neural network model for the automatic classification of nucleosome forming sequences has been proposed. The proposed network is able to exploit the information about nucleotides position in a sequence, by using a mix of convolutional and recurrent layers. The effectiveness of the proposed method is proved in terms of Sensitivity, specificity, accuracy and AUC values, for which it pushes forward the state of the art in almost all the considered datasets. Anyway, we think that the major advantage of what we are proposing, is that it does not make any use of any a priori information, or feature extraction or selection step, about the features that are relevant for the classification of nucleosomes. It automatically learns the task by using the training set of labelled sequences, using the *character-level one-hot encoding* as input representation. Actually, at least 300 of experimental datasets of genome-wide nucleosome occupancy profiles determined in the different cell type of about ten species are currently available [48]. Taking into consideration this actual scenario, we think that the method we propose, due to this precision, could be very useful in the study of the difference in nucleosome organization between different species.

## Abbreviations

AUC: Area under the ROC curve
CE: Caenorhabditis elegans
CNNs: Convolutional neural networks
DLNN: Deep learning neural networks
DM: Drosophila melanogaster
HM: Homo sapiens
LC: Largest chromosome
LSTM: Long short-term memory
PM: Promoter
ReLU: rectified linear units
RNNs: Recurrent neural networks
ROC: Receiver operating characteristic
WG: Whole genome
YS: Yeast
5U: 5’UTR exon region
